# Role of human heterogeneous nuclear ribonucleoprotein C1/C2 in dengue virus replication

**DOI:** 10.1186/s12985-014-0219-7

**Published:** 2015-02-06

**Authors:** Thanyaporn Dechtawewat, Pucharee Songprakhon, Thawornchai Limjindaporn, Chunya Puttikhunt, Watchara Kasinrerk, Sawanan Saitornuang, Pa-thai Yenchitsomanus, Sansanee Noisakran

**Affiliations:** Division of Molecular Medicine, Office of Research and Development, Faculty of Medicine Siriraj Hospital, Mahidol University, Bangkok, 10700 Thailand; Graduate Program in Immunology, Department of Immunology, Faculty of Medicine Siriraj Hospital, Mahidol University, Bangkok, 10700 Thailand; Department of Anatomy, Faculty of Medicine Siriraj Hospital, Mahidol University, Bangkok, 10700 Thailand; Medical Biotechnology Research Unit, National Center for Genetic Engineering and Biotechnology, National Science and Technology Development Agency, Bangkok, 10700 Thailand; Division of Dengue Hemorrhagic Fever Research Unit, Office of Research and Development, Faculty of Medicine Siriraj Hospital, Mahidol University, Bangkok, 10700 Thailand; Division of Clinical Immunology, Department of Medical Technology, Faculty of Associated Medical Sciences, Chiang Mai University, Chiang Mai, 50200 Thailand; Biomedical Technology Research Center, National Center for Genetic Engineering and Biotechnology, National Science and Technology Development Agency, Chiang Mai, 50200 Thailand

**Keywords:** hnRNP C1/C2, Dengue virus, siRNA transfection, Virus replication

## Abstract

**Background:**

Host and viral proteins are involved in dengue virus (DENV) replication. Heterogeneous ribonucleoprotein (hnRNP) C1/C2 are abundant host cellular proteins that exhibit RNA binding activity and play important roles in the replication of positive-strand RNA viruses such as poliovirus and hepatitis C virus. hnRNP C1/C2 have previously been shown to interact with vimentin and viral NS1 in DENV-infected cells; however, their functional role in DENV replication is not clearly understood. In the present study, we investigated the role of hnRNP C1/C2 in DENV replication by using an *in vitro* model of DENV infection in a hepatocyte cell line (Huh7) and siRNA-mediated knockdown of hnRNP C1/C2.

**Methods:**

Huh7 cells were transfected with hnRNP C1/C2-specific siRNA or irrelevant siRNA (control) followed by infection with DENV. Mock and DENV-infected knockdown cells were processed for immunoprecipitation using hnRNP C1/C2-specific antibody or their isotype-matched control antibody. The immunoprecipitated samples were subjected to RNA extraction and reverse transcriptase polymerase chain reaction (RT-PCR) for detection of DENV RNA. In addition, the knockdown cells harvested at varying time points after the infection were assessed for cell viability, cell proliferation, percentage of DENV infection, amount of viral RNA, and viral E and NS1 expression. Culture supernatants were subjected to focus forming unit assays to determine titers of infectious DENV. DENV luciferase reporter assay was also set up to determine viral translation.

**Results:**

Immunoprecipitation with the anti-hnRNP C1/C2 antibody and subsequent RT-PCR revealed the presence of DENV RNA in the immunoprecipitated complex containing hnRNP C1/C2 proteins. Transfection with hnRNP C1/C2-specific siRNA resulted in a significant reduction of hnRNP C1/C2 mRNA and protein levels but did not induce cell death during DENV infection. The reduced hnRNP C1/C2 expression decreased the percentage of DENV antigen-positive cells as well as the amount of DENV RNA and the relative levels of DENV E and NS1 proteins; however, it had no direct effect on DENV translation. In addition, a significant reduction of DENV titers was observed in the supernatant from DENV-infected cells following the knockdown of hnRNP C1/C2.

**Conclusions:**

Our findings suggest that hnRNP C1/C2 is involved in DENV replication at the stage of viral RNA synthesis.

## Background

Dengue virus (DENV) infection poses a major public health threat that affects over 40% of the world’s population [1]. Infection with any of four serotypes of DENV (i) can be asymptomatic; (ii) cause a self-limiting, mild febrile illness known as dengue fever; or (iii) cause life-threatening illnesses called dengue hemorrhagic fever (DHF) and dengue shock syndrome (DSS) [2,3]. At present, the mechanism of DENV infection that leads to pathogenesis of DHF/DSS remains unclear.

DENV is a member of the flaviviruses in the family *Flaviviridae*. It is a positive-sense, single-stranded, enveloped RNA virus that has previously been reported to enter different types of host cells, for example, dendritic cells, monocytes, endothelial cells and hepatocytes, through receptor-dependent mechanisms or antibody-dependent enhancement [4-7]. DENV polyprotein is encoded by the positive-strand viral RNA genome and processed in DENV-infected cells by viral and cellular proteases to generate three structural proteins (capsid, C; pre-membrane, prM; and envelope, E) and seven nonstructural proteins (NS1, NS2A, NS2B, NS3, NS4A, NS4B and NS5) [8,9]. Interactions of these viral proteins with several host cellular proteins play important roles in the entry, replication, assembly and egress of DENV, as well as cell signaling and immune escape from host responses during DENV infection, as reported previously [10-13].

hnRNP C1/C2 are abundant host cellular proteins among at least 20 members (hnRNP A-U) of the hnRNP family [14,15] that share common characteristics, but still possess a variety of domain compositions and functional properties. hnRNP C1/C2 differ by 13-amino acid residues after glycine 106 or serine 107 as a result of alternative mRNA splicing, and the longer isoform (C2) comprises ~25% of the total hnRNP C in the cells [16,17]. Both the hnRNP C isoforms contain an RNA recognition motif and a basic leucine zipper-like RNA-binding motif that bind RNA in the form of heterotetramers with three molecules of C1 and one molecule of C2 [18,19]. hnRNP C1/C2 are involved in mRNA biogenesis, transport and stability, as well as protein translation [14,15]. Normally, hnRNP C1/C2 reside mainly in the nucleus [20]. However, certain cellular conditions (e.g., apoptosis, mitosis and virus infection) induce hnRNP C1/C2 to translocate from the nucleus to the cytoplasm [21-25]. hnRNP C1/C2 were previously identified as DENV NS1-interacting host partners but the role of their association remained undefined [26]. Additionally, hnRNP C1/C2 have been found to interact with vimentin, an intermediate filament supporting cell integrity, which is recruited to the perinuclear region with dense structural rearrangement during DENV infection, and is necessary for the infection process [27-29].

To explore the potential involvement of hnRNP C1/C2 in DENV replication, we employed an *in vitro* study using the hepatocyte Huh7 cell line for DENV infection and specific siRNA-mediated gene knockdown. Effects of hnRNP C1/C2 knockdown on different events in DENV infection and replication have been investigated.

## Results

### Association of hnRNP C1/C2 proteins with DENV RNA

**Figure 1:**
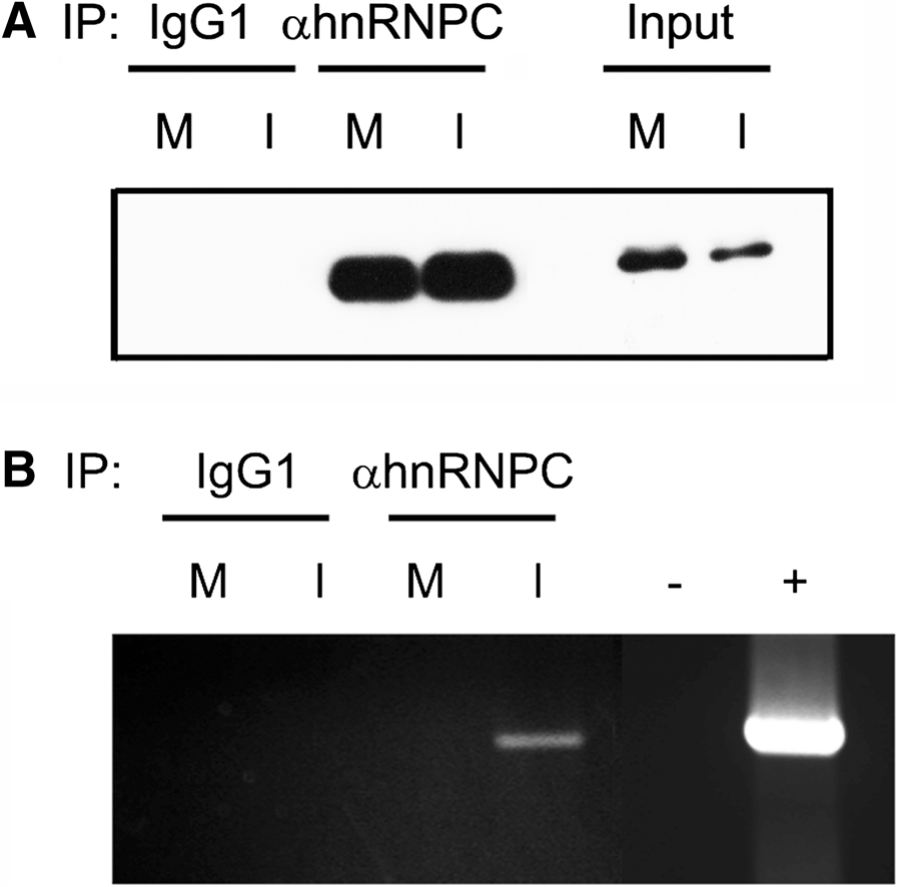
**Association of hnRNP C1/C2 proteins with dengue viral RNA.** Mock-infected (M) and DENV-infected (I) cells were subjected to immunoprecipitation using anti-hnRNPC1/C2 (αhnRNP C) monoclonal antibody and their isotype-matched control antibody (IgG1). **(A)** Immunoprecipitated proteins were analyzed by immunoblotting using hnRNP C1/C2-specific antibody. Mock and DENV-infected cell lysates prior to immunoprecipitation served as controls (input). **(B)** RNA was extracted from the immunoprecipitated samples and used as a template for RT-PCR with a primer pair specific for DENV NS1 region. PCRs performed in parallel in the absence of cDNA and in the presence of pcDNAhygro containing DENV NS1 gene were included as negative (−) and positive (+) controls, respectively. Results are representative of three independent experiments with similar outcome.

To address a possibility that hnRNP C1/C2, RNA-binding proteins, might associate with viral RNA in DENV-infected cells, immunoprecipitation of mock-infected and DENV-infected cell lysates was performed using anti-hnRNP C1/C2 specific antibody or its isotype-matched control antibody. Immunoprecipitated samples were assayed for the presence of hnRNP C1/C2 proteins by immunoblotting analysis and also subjected to RNA extraction and subsequent RT-PCR for detection of DENV NS1 amplicon. Prior to immunoprecipitation, immunoblotting analysis demonstrated that hnRNP C1/C2 were present in mock-infected and DENV-infected cell lysates (Figure 1A, Input: M and I). Immunoprecipitation with anti-hnRNP C1/C2 antibody allowed for enrichment of hnRNP C1/C2 proteins in mock-infected and DENV-infected samples (Figure 1A, αhnRNP C: M and I), while no specific protein band was discernible following immunoprecipitation with isotype-matched control antibody (Figure 1A, IgG1: M and I). Further analysis by RT-PCR revealed that an amplicon band corresponding to DENV NS1 region was detected in DENV-infected samples immunoprecipitated with anti-hnRNP C1/C2 antibody (Figure 1B, αhnRNP C: I). Mock-infected samples immunoprecipitated with the same antibody did not yield any amplicon band (Figure 1B, αhnRNP C: M). In addition, no specific band was evident in mock-infected and DENV-infected samples immunoprecipitated with isotype-matched control antibody (Figure 1B, IgG1: M and I). The results indicated the specific detection of viral RNA in the context of hnRNP C1/C2 complexes in DENV-infected cells.

### Knockdown of hnRNP C1/C2 by specific siRNA transfection

**Figure 2:**
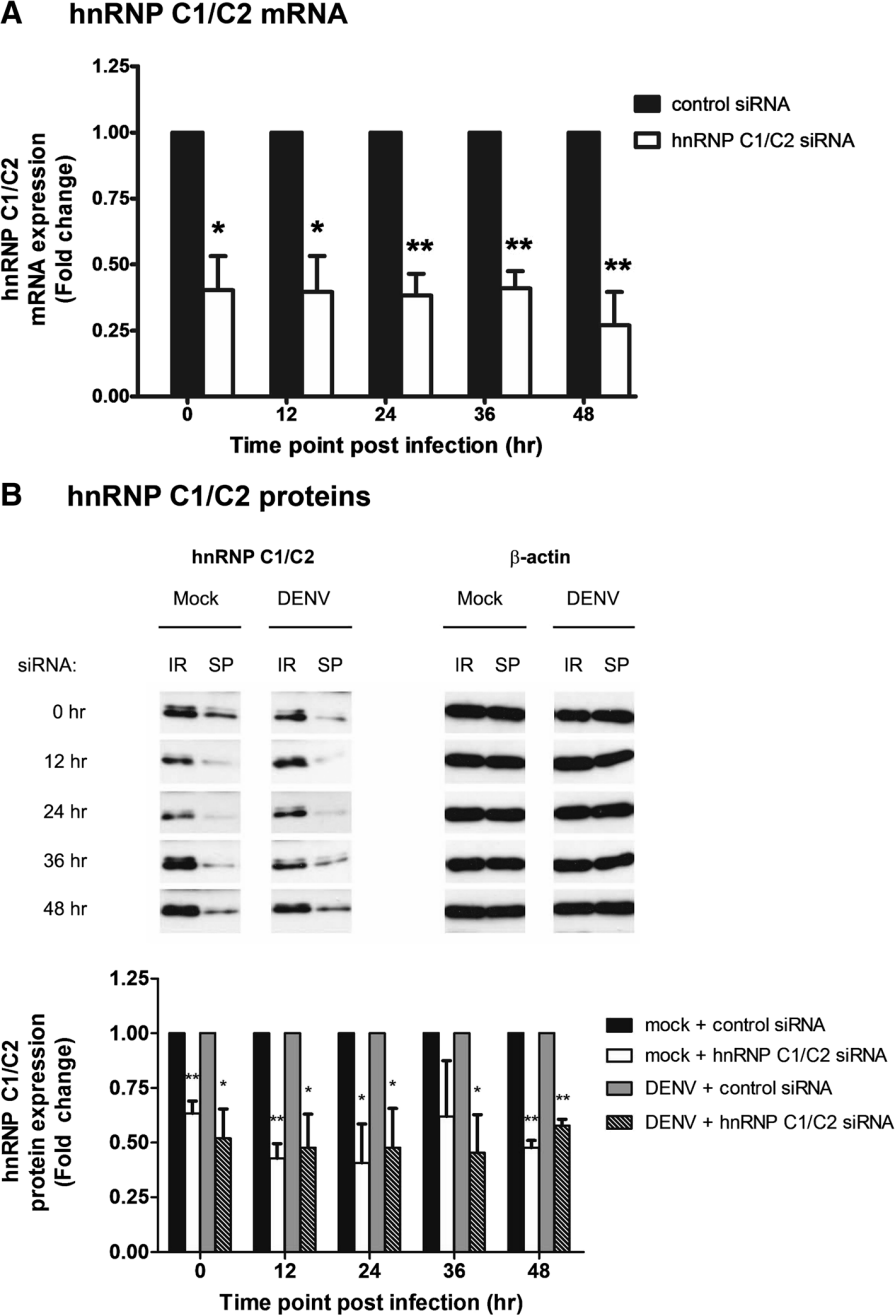
**siRNA-mediated knockdown of hnRNP C1/C2 mRNA and proteins. (A)** Huh7 cells transfected with hnRNP C1/C2-specific or control siRNA were infected with DENV at MOI 0.03. Cells were collected at 0, 12, 24, 36 and 48 h post-infection and subjected to total RNA extraction and subsequent reverse transcription and real-time PCR for determination of hnRNP C1/C2 and β-actin (internal control) mRNA expression. Relative hnRNP C1/C2 mRNA expression was obtained by normalization with β-actin mRNA expression in siRNA-transfected cells following DENV infection. Fold change of the relative hnRNP C1/C2 mRNA expression between the specific siRNA and control siRNA-transfected samples was compared. Data represent mean and SEM of three independent experiments. Asterisks indicate statistically significant differences (*p < 0.05; **p < 0.01) in hnRNP C1/C2 levels between the specific siRNA-transfected and control siRNA-transfected groups by unpaired *t* test. **(B)** Huh7 cells were transfected with hnRNP C1/C2-specific siRNA (SP) or irrelevant negative control siRNA (IR), followed by mock or DENV infection at MOI 0.03. Cells were collected at the indicated time points post-infection and subjected to immunoblotting analysis using monoclonal antibodies specific against human hnRNP C1/C2 and β-actin (internal protein control). Immunoblotting analysis of hnRNP C1/C2 and β-actin proteins in mock-infected and DENV-infected cells was shown as representative of three independent experiments (top panel). The intensity of hnRNP C1/C2 bands in both IR and SP samples was first normalized to that of β-actin in the same set of samples. The obtained relative intensity of hnRNP C1/C2 in the SP samples was then normalized to that in the IR samples. Results show fold change of relative hnRNP C1/C2 protein expression in the SP samples compared with the corresponding IR samples (bottom panel). Data represent mean and SEM of three independent experiments. Asterisks indicate statistically significant differences (*p < 0.05; **p < 0.01) in relative expression of hnRNP C1/C2 proteins between the specific siRNA-transfected and control siRNA-transfected samples by unpaired *t* test.

Whether hnRNP C1/C2 proteins associating with viral RNA are involved in the DENV infection process was addressed using siRNA transfection to knockdown hnRNP C1/C2 prior to DENV infection. Initially, we tested the effect of siRNA transfection on hnRNP C1/C2 mRNA expression. Huh7 cells were transfected with either hnRNP C1/C2-specific siRNA or irrelevant control siRNA and subsequently infected with DENV. The cells were harvested post-infection to determine the expression of hnRNP C1/C2 and β-actin (internal control) mRNA using real time RT-PCR. Relative levels of hnRNP C1/C2 mRNA expression was significantly decreased by approximately 60–70% in hnRNP C1/C2 siRNA-transfected cells compared with control cells at all time points tested (Figure 2A). This indicated the efficiency of hnRNP C1/C2 mRNA knockdown during DENV infection.

To confirm whether levels of hnRNP C1/C2 proteins are reduced following specific siRNA transfection, uninfected cells (mock control) or DENV-infected cells that had been transfected with either hnRNP C1/C2-specific siRNA or irrelevant control siRNA were subjected to immunoblotting analysis using specific antibody against hnRNP C1/C2 and β-actin proteins. Immunoblotting showed that transfection with hnRNP C1/C2-specific siRNA resulted in decreased expression of hnRNP C1/C2 protein bands in mock-infected and DENV-infected cells compared with control siRNA transfection. Equivalent expression of β-actin protein bands (internal control) was observed in the same set of samples following transfection with either siRNA (Figure 2B, top panel). Normalization of hnRNP C1/C2 to β-actin protein demonstrated that relative expression of hnRNP C1/C2 proteins was reduced by 40–60% during the whole study period in both mock-infected and DENV-infected cells transfected with hnRNP C1/C2-specific siRNA compared with control siRNA-transfected cells (Figure 2B, bottom panel). This hnRNP C1/C2 knockdown effect could still be observed even at 48 h post-infection (Figure 2B).

### Effects of hnRNP C1/C2 knockdown on cell viability and proliferation

**Figure 3:**
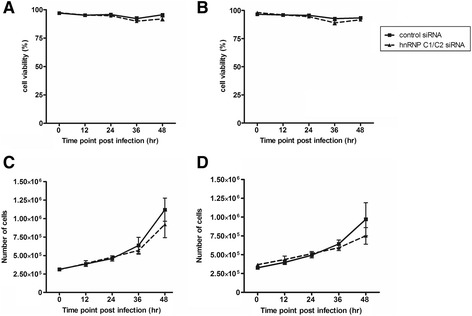
**Viability and proliferation of mock and DENV-infected Huh7 cells following hnRNP C1/C2 knockdown.** Huh7 cells were transfected with either hnRNP C1/C2-specific siRNA or irrelevant negative control siRNA followed by mock or DENV infection at MOI 0.03. Cells were collected at 0, 12, 24, 36 and 48 h post-infection and subjected to propidium iodide (PI) staining and trypan blue exclusion dye assay. Results are shown as follows: **(A)** the percentage viability of mock-infected cells; **(B)** the percentage viability of DENV-infected cells; **(C)** the total number of viable mock-infected cells; and **(D)** the total number of viable DENV-infected cells. Data represent mean and SEM of three independent experiments.

To determine whether knockdown of hnRNP C1/C2 proteins affects cell viability and proliferation, mock-infected and DENV-infected cells that had been transfected with hnRNP C1/C2-specific siRNA or irrelevant control siRNA were assessed for the percentage of cell viability and the total cell number at varying time points post infection as described in the Materials and Methods. Mock-infected and DENV-infected knockdown cells transfected with either siRNA showed similar percentages of cell viability ranging from 90% to 97% within 48 h of the study period (Figure 3A and B). This suggested that knockdown of hnRNP C1/C2 proteins did not induce cell death following DENV infection (Figure 3A and B). Regardless of the type of siRNA used for transfection, both mock-infected and DENV-infected knockdown cells increased over time with the same kinetic pattern of proliferation during 36 h post infection (Figure 3C and D). Although the total number of mock-infected and DENV-infected cells, which had been transfected with hnRNP C1/C2-specific siRNA, seemed to be relatively lower than that transfected with control siRNA at 48 h post infection, no statistically significant difference in cell proliferation was observed at this time point (Figure 3C and D). Thus, knockdown of hnRNP C1/C2 proteins had no profound effect on proliferation of mock-infected and DENV-infected cells within the study period tested.

### Effects of hnRNP C1/C2 knockdown on DENV RNA and protein syntheses

**Figure 4:**
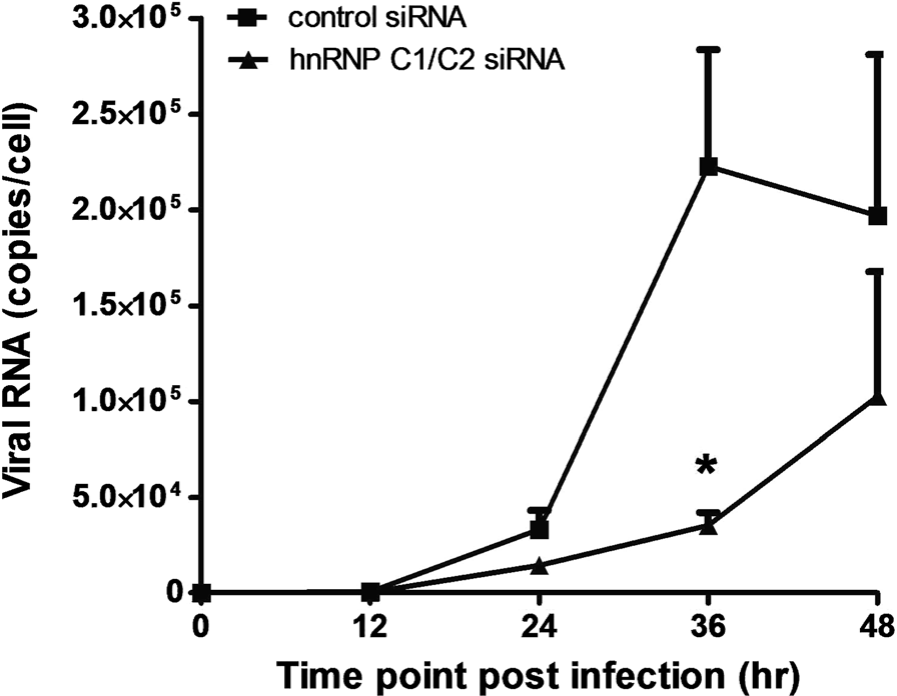
**Effect of hnRNP C1/C2 knockdown on DENV RNA synthesis.** Huh7 cells transfected with hnRNP C1/C2-specific or control siRNA were infected with DENV at MOI 0.03. Cells were collected at 0, 12, 24, 36 and 48 h post-infection and subjected to total RNA extraction and subsequent reverse transcription and real-time PCR for determination of DENV RNA expression. The amount of DENV RNA in virus-infected cells was reported as viral RNA copies per cell. Data represent mean and SEM of three independent experiments. Asterisks indicate statistically significant differences (*p < 0.05) in DENV viral RNA levels between the specific siRNA-transfected and control siRNA-transfected groups by unpaired *t* test.

To investigate possible effects of hnRNP C1/C2 knockdown on specific events in DENV replication, control and hnRNP C1/C2 siRNA-transfected cells were infected with DENV and harvested after infection to determine levels of DENV E RNA, the percentage of DENV infection and levels of DENV NS1 and E proteins, using real-time RT-PCR, immunofluorescence staining and immunoblotting analysis, respectively. Real-time RT-PCR demonstrated that DENV RNA was detected in control and hnRNP C1/C2 siRNA-transfected cells in a time-dependent manner following DENV infection (Figure 4). The levels of DENV RNA in the control siRNA-transfected cells rapidly increased and peaked within 36 h post-infection. However, the kinetics of DENV RNA appeared to be delayed in hnRNP C1/C2 siRNA-transfected cells and its expression was lower than that in the control cells at 24, 36 and 48 h post-infection. At 36 h post-infection, there was an approximately six-fold difference in DENV RNA levels between the control and hnRNP C1/C2 siRNA-transfected cells. Although a statistically significant difference in DENV RNA levels between the control and hnRNP C1/C2 siRNA-transfected cells was not observed at every time point tested, a tendency of reducing DENV RNA levels following specific siRNA transfection suggested the interference of hnRNP C1/C2 knockdown on DENV RNA synthesis.

**Figure 5:**
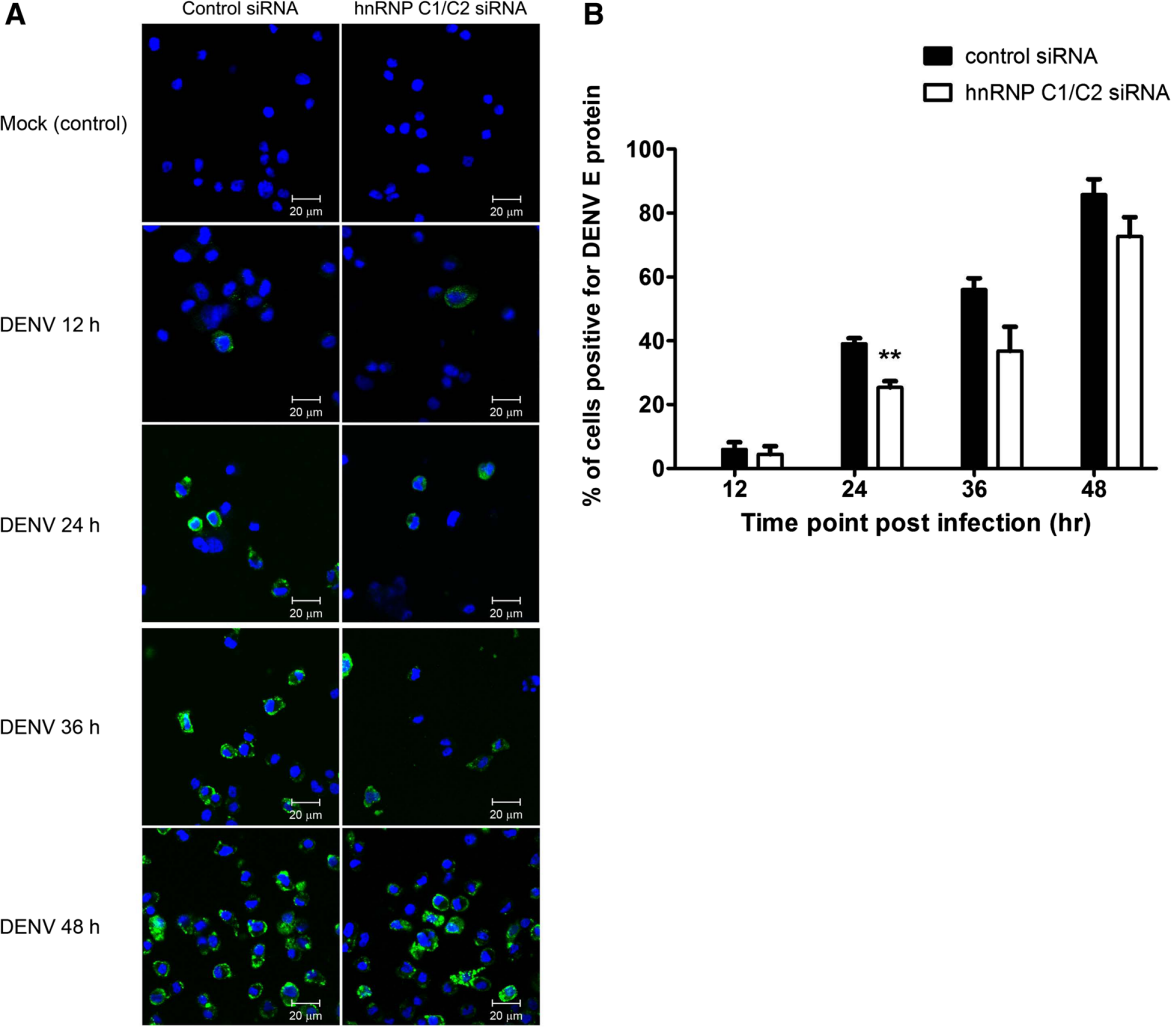
**Effect of hnRNP C1/C2 knockdown on the extent of DENV infection.** Huh7 cells transfected with hnRNP C1/C2-specific siRNA or control siRNA were infected with DENV at MOI 0.03 or left uninfected (mock control). The cells were collected at 12, 24, 36 and 48 h post-infection and subjected to immunofluorescence staining for DENV E antigen. The extent of DENV infection was determined based on the enumerated numbers of DENV E antigen-positive cells. **(A)** Representative images of immunofluorescently stained cells from three independent experiments. Images of mock-infected cells at 24 h post-infection and DENV-infected cells at 12, 24, 36 and 48 h post-infection are shown. Dengue E antigen (green); nucleus (blue); magnification (63×). **(B)** Percentage infection is shown as mean and SEM of three independent experiments. Asterisks indicate statistically significant differences (**p < 0.01) in the percentage DENV infection between the specific siRNA-transfected and control siRNA-transfected groups by unpaired *t* test.

A parallel set of siRNA-transfected cells was subjected to immunofluorescence staining for DENV E antigen and assessed for the extent of DENV infection. Only 4–6% of control and hnRNP C1/C2 siRNA-transfected cells expressed detectable DENV E antigen at 12 h post-infection. However, the number of DENV E antigen-expressing cells rapidly increased over time following DENV infection (Figure 5A and B). hnRNP C1/C2 siRNA-transfected cells yielded a lower extent of DENV infection than control siRNA-transfected cells, namely 25%, 37% and 73% versus 39%, 56% and 86% of observed cells showing DENV E expression at 24, 36 and 48 h post-infection, respectively (Figure 5A and B). This decrease in the proportion of DENV E-positive cells reflected a consequence of hnRNP C1/C2 knockdown on DENV infection.

**Figure 6:**
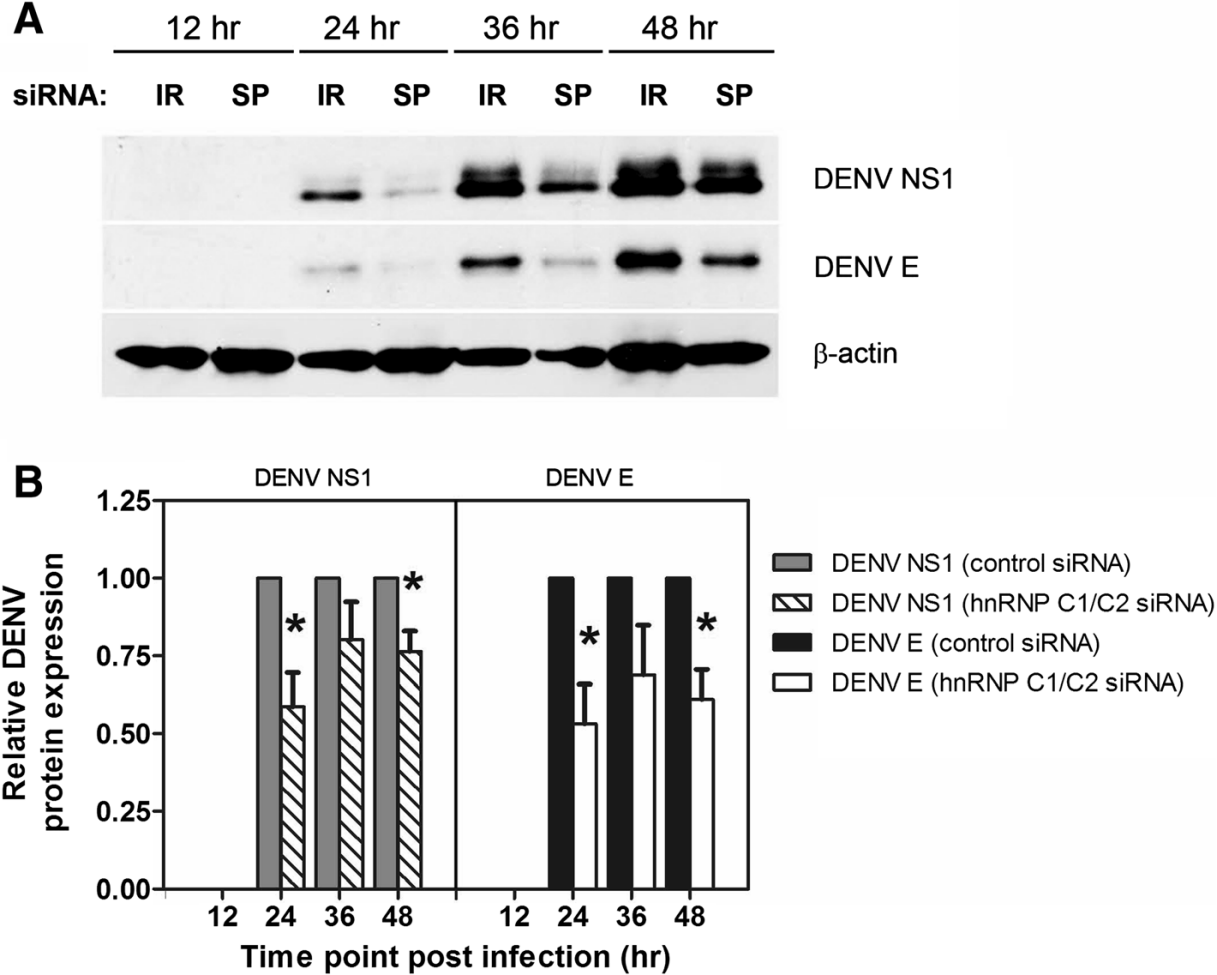
**Effect of hnRNP C1/C2 knockdown on DENV protein expression.** Huh7 cells transfected with hnRNP C1/C2-specific siRNA (SP) or irrelevant negative control siRNA (IR) were infected with DENV at MOI 0.03. Cells were collected at 12, 24, 36 and 48 h post-infection and subjected to immunoblotting analysis using monoclonal antibodies specific against DENV NS1, DENV E and β-actin (internal control). Three independent experiments were performed. **(A)** Protein bands of DENV NS1, DENV E and β-actin in DENV-infected cells transfected with specific siRNA (SP) or control siRNA (IR). **(B)** The intensity of DENV NS1 and E bands in both IR and SP samples was first normalized to that of β-actin bands in the same set of samples. The obtained relative intensity of DENV NS1 and E in the SP samples was then normalized to that in the IR samples. Results show fold change of relative DENV NS1 and E protein expression in the SP samples compared with the corresponding IR samples. Asterisks indicate statistically significant differences (*p < 0.05) in DENV NS1 and E protein expression between the specific siRNA-transfected and control siRNA-transfected groups by unpaired *t* test.

Further analysis of DENV NS1 and E protein levels in control and hnRNP C1/C2 siRNA-transfected cells using immunoblotting showed that both DENV protein bands were initially visible at 24 h post-infection, and their expression increased over time in both the siRNA-transfected samples after DENV infection (Figure 6A). The intensity of the DENV NS1 and E protein bands was lower in hnRNP C1/C2 siRNA-transfected cells (SP) than the control cells (IR) at all time points (Figure 6A). After normalization with β-actin (internal control), relative levels of DENV NS1 and E protein expression in hnRNP C1/C2 siRNA-transfected cells decreased by 20–40% and 30–50%, respectively, compared with the control cells at 24–48 h post-infection (Figure 6B). At 48 h post infection, although relative levels of DENV NS1 and E protein expression in hnRNP C1/C2 siRNA-transfected cells were significant different from that in the control cells (Figure 6); the percentage of DENV E antigen-expressing cells transfected with either siRNA did not show statistically significant difference (Figure 5). This implied that hnRNP C1/C2 knockdown may have no significant impact on the number of DENV-infected cells when encountering multiple rounds of virus replication, but it could still decrease the extent of viral protein expression significantly. Altogether, our findings based on real-time RT-PCR, immunofluorescence staining and immunoblotting analysis suggested that hnRNP C1/C2 knockdown had effects on viral RNA and protein syntheses during DENV replication.

**Figure 7:**
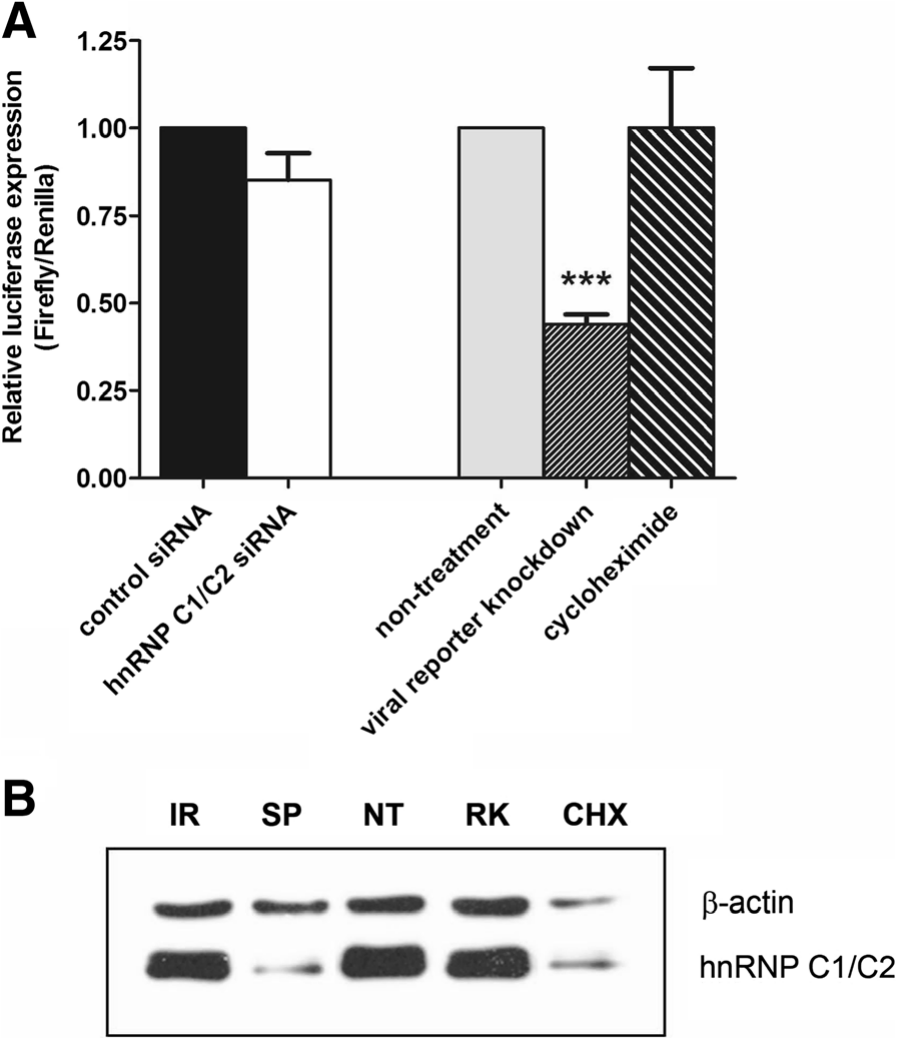
**Effect of hnRNP C1/C2 knockdown on DENV protein translation.** Huh7 cells transfected with hnRNP C1/C2-specific siRNA (SP) or irrelevant negative control siRNA (IR) were subjected to co-transfection with pGL3-DENV2-5′UTR-72ntC-Fluc-3′UTR construct (viral reporter) and pRL-SV40 construct (internal control reporter). At 12 h after reporter RNA transfection, cells were harvested and assessed for firefly and *Renilla* luciferase activities. In parallel, reporter RNA-transfected cells that were treated with DENV capsid-specific siRNA to knockdown viral reporter RNA template used for viral translation (RK) or with cycloheximide to inhibit overall protein translation (CHK) were set up as positive controls in this assay for inhibition of protein translation, compared with non-treated cells (NT). **(A)** Relative luciferase expression specifically indicated DENV protein translation. In each sample, relative luminescence units of firefly luciferase activity were normalized to that of *Renilla* luciferase activity. Normalized luciferase signals of control siRNA-transfected cells and non-treated cells were set to 1. Relative luciferase expression in hnRNP C1/C2-specific siRNA was compared to that in control siRNA-transfected cells whereas relative luciferase expression in viral reporter knockdown cells and cycloheximide-treated cells was compared to that in non-treated cells. Data represent mean and SEM of three independent experiments. Asterisks indicate statistically significant difference (***p < 0.0001) in relative luciferase expression between viral reporter knockdown cells and non-treated cells by unpaired *t* test. **(B)** Immunoblotting analysis was performed to confirm the expression of hnRNP C1/C2 and β-actin proteins in each sample. Results are representative of three independent experiments with similar outcome.

To further investigate whether the effect of hnRNP C1/C2 knockdown on viral protein synthesis is due to direct inhibition of DENV translation, control and hnRNP C1/C2 siRNA-transfected cells were co-transfected with DENV reporter RNA and internal control reporter RNA. In parallel, DENV reporter RNA-transfected cells that were treated with DENV capsid-specific siRNA to knockdown DENV reporter RNA template used for viral translation or with cycloheximide to inhibit overall protein translation served as positive controls for inhibition of protein translation. Firefly and *Renilla* luciferase activities were measured at 12 h after reporter RNA transfection and relative luciferase expression was specifically indicative of DENV protein translation. In addition, immunoblotting analysis was performed to confirm the levels of hnRNP C1/C2 and β-actin expression in the same set of samples. As expected, DENV reporter RNA knockdown cells showed approximately 60% specific inhibition of viral protein translation (Figure 7A) and expressed similar levels of hnRNP C1/C2 and β-actin proteins, compared with non-treated cells (Figure 7B, NT and RK). Treatment with cycloheximide appeared to reduce both viral and host protein translation in our assay as decreased levels of both firefly and *Renilla* luciferase activities were observed (data not shown) and, therefore, no specific inhibition of viral protein translation was detected in cycloheximide-treated cells when compared with non-treated cells (Figure 7A). A marked reduction of hnRNP C1/C2 and β-actin expression observed in the same sample also confirmed this notion (Figure 7B, CHX compared with NT). In contrast to these positive controls, transfection with hnRNP C1/C2-specific siRNA did not result in specific inhibition of viral protein translation (Figure 7A) since levels of both firefly and *Renilla* luciferase activities remained unchanged (data not shown), despite a specific decrease in hnRNP C1/C2 protein expression detected (Figure 7B, SP), when compared to control siRNA transfection (Figure 7A and B, IR). These findings implied that hnRNP C1/C2 knockdown had no direct influence on DENV protein translation.

### Effect of hnRNP C1/C2 knockdown on infectious DENV production

**Figure 8:**
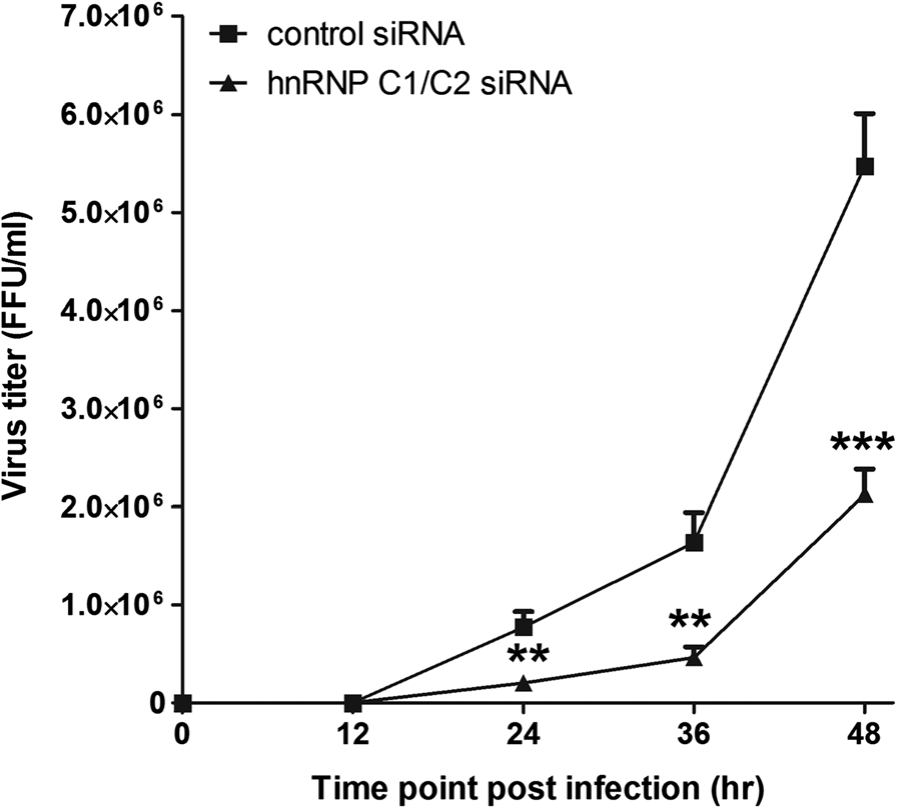
**Effect of hnRNP C1/C2 knockdown on release of infectious DENV.** Huh7 cells transfected with hnRNP C1/C2-specific siRNA or control siRNA were infected with DENV at MOI 0.03. Supernatant from DENV-infected culture was collected at 0, 12, 24, 36 and 48 h post-infection and assessed for titers of infectious DENV by FFU assay. Results are derived from three independent experiments. Asterisks indicate statistically significant differences (**p < 0.01; ***p < 0.005) in DENV titers between the specific siRNA-transfected and control siRNA-transfected groups by unpaired *t* test.

Further study was carried out to examine whether hnRNP C1/C2 knockdown affected the production of infectious DENV. Supernatants from control and hnRNP C1/C2 siRNA-transfected cell cultures were collected and assessed for infectious virus titers by FFU assay. Extracellular infectious DENV was detectable at low levels (<10 FFU/ml) in culture supernatants collected at 12 h post-infection from control and hnRNP C1/C2 siRNA-transfected cultures, and increased continuously thereafter in both cultures (Figure 8). However, DENV titers in the hnRNP C1/C2 siRNA-transfected culture were significantly lower than in the control siRNA-transfected culture by 3–4 fold at 24–48 h post-infection. A reduction of infectious DENV production in the cultures following specific siRNA transfection confirmed the interference of hnRNP C1/C2 knockdown on DENV replication.

## Discussion

hnRNP C1/C2 are RNA-binding host cellular proteins that play important roles in replication of certain positive-strand RNA viruses such as poliovirus and hepatitis C virus (HCV) [30-33]. Nevertheless, their functional contribution to DENV replication remains largely unexplored. The present study used an *in vitro* model of Huh7 cells for DENV infection and specific siRNA-mediated gene knockdown to investigate the potential involvement of hnRNP C1/C2 in the process of DENV infection. We demonstrated an association between hnRNP C1/C2 and viral RNA in DENV-infected Huh7 cells. A significant knockdown of hnRNP C1/C2 proteins following specific siRNA transfection affected DENV infection by decreasing viral RNA replication, viral protein synthesis and subsequent production of infectious virus.

hnRNP C1/C2 are abundant host cellular proteins found predominantly in the nucleus, but they can translocate to the cytoplasm under certain conditions [20-25]. Active export of hnRNP C1/C2 to the cytoplasm was triggered by tumor necrosis factor-α or phorbol 12-myristate 13-acetate (PMA)-mediated apoptosis through activation of Rho-associated kinase [22]. The presence of hnRNP C1/C2 in the cytoplasm was also observed during mitosis upon nuclear membrane breakdown in late G2/M phase of the cell cycle [25]. Additionally, cytoplasmic re-localization of hnRNP C1/C2 has been found in rhinovirus, poliovirus and vesicular stomatitis virus infections, through inhibition of nuclear import and changes in the composition of nuclear pore complexes [21,23] or enhanced nuclear export of the proteins [24]. A previous study of DENV infection demonstrated the presence of hnRNP C1/C2 predominantly in the nuclear fraction, and to a lesser degree in the cytoplasmic fraction of virus-infected EA.hy926 cells, using a subcellular fractionation assay [28]. This finding was consistent with the detection of hnRNP C1/C2 in the cytoplasm by immunofluorescence staining in DENV-infected HEK 293T cells [26] and DENV-infected Huh7 cells (data not shown). However, no difference in the pattern of subcellular localization of hnRNP C1/C2 between mock-infected and DENV-infected cells was observed [26].

Specific siRNA transfection resulted in a significant decrease in hnRNP C1/C2 at both mRNA and protein levels (Figure 2A and B). As demonstrated by immunofluorescence staining of DENV E antigen, the hnRNP C1/C2 knockdown reduced the percentage of cells expressing this viral protein (Figure 5). This effect was unlikely due to a direct hindrance at the initial step of DENV binding and entry into the cells, because hnRNP C1/C2 were undetectable on the cell surface (data not shown). Rather, it probably resulted from multiple downstream processes within the DENV replication cycle that led to a signification decrease in DENV production (Figure 8). Using real-time RT-PCR, delayed kinetics of viral RNA synthesis were observed in DENV-infected cells following hnRNP C1/C2 knockdown, particularly with a significant reduction of viral RNA accumulation at 36 h post-infection (Figure 4). This suggested a potential contribution of hnRNP C1/C2, in association with viral RNA, to facilitate the RNA replication process of DENV. Similar findings on the role of hnRNP C1/C2 in virus replication have been reported in other positive-strand RNA viruses such as poliovirus and HCV [30-33]. hnRNP C1/C2 have been shown to interact with 5′- and 3′-ends of poliovirus negative-strand RNA intermediate and with poliovirus protein precursors, which are essential for virus replication; that is, 3CD (a precursor of viral RNA-dependent RNA polymerase), P2 and P3 (precursors of nonstructural proteins), hence promoting viral replication complex assembly and viral RNA synthesis of poliovirus [31-33]. Moreover, hnRNP C1/C2, accompanied by polypyrimidine-tract binding protein (PTB or hnRNP I), bind to the pyrimidine-rich region within the 3′-untranslated region (UTR) of HCV RNA and initiate and/or regulate HCV replication [30]. The detailed mechanism whereby hnRNP C1/C2 associate with viral RNA in DENV-infected cells is not known. It might be that this interaction functions, in concert with other viral and host proteins, to assist the structural formation of the viral replication complex required for DENV replication.

hnRNP C1/C2 have previously been found to interact with vimentin and viral NS1 in DENV-infected cells and disruption of vimentin results in reduced cell-associated DENV NS1 expression and DENV production [28]. In support of these findings, the present study revealed that hnRNP C1/C2 knockdown diminished the expression of DENV NS1 and E proteins in DENV-infected cells (Figure 6). Whether the hnRNP C1/C2 knockdown may alter the presence of vimentin in the intracellular milieu is of interest but has not been assessed in our study. It was possible that hnRNP C1/C2 proteins may support viral RNA stability, leading to efficient DENV protein expression. Previous studies have demonstrated that binding of hnRNP C1/C2 with 3′-UTR of amyloid precursor protein mRNA and urokinase receptor mRNA increase the stability of mRNAs [34,35]. Furthermore, hnRNP C1/C2 function as translational modulators to regulate protein expression, most likely through their specific binding to polyuridine-rich regions of RNA, some of which may have internal ribosome entry site (IRES) activity [14]. Interactions of hnRNP C1/C2 with IRES of c-myc mRNA, c-cis mRNA, upstream of N-Ras (UNR) mRNA and X-linked inhibitor of apoptosis (XIAP) mRNA stimulate IRES-mediated translation, thus resulting in increased protein expression [25,36-38]. Unlike poliovirus and HCV, 5′-UTR of DENV genome does not possess IRES activity, and translation of DENV occurs by non-IRES-mediated mechanisms through canonical cap-dependent and noncanonical cap-independent processes [39]. In our study, knockdown of hnRNP C1/C2 did not show a direct effect on DENV protein translation as demonstrated by the DENV luciferase reporter assay (Figure 7A and B). As a consequence, the reduced viral protein expression following knockdown of hnRNP C1/C2 observed in this study likely resulted from a reduction of viral RNA templates generated during DENV replication rather than direct influence on viral translation.

Following viral RNA replication and protein translation, assembly of DENV particles occurs in the ER. Immature viruses are subsequently transported along the secretory pathway to the trans-Golgi network for further modification, before release into the extracellular milieu [9,40]. In our study, determination of DENV titers in the culture supernatant demonstrated a significant reduction of infectious virus production from DENV-infected cells upon hnRNP C1/C2 knockdown (Figure 8). This may have been the consequence of decreased levels of viral RNA and protein syntheses in DENV-infected knockdown cells. The suppressive effects of hnRNP C1/C2 knockdown on viral RNA replication and protein expression and infectious virus production were unlikely to have resulted from induction of cell death and inhibition of cell proliferation owing to hnRNP C1/C2 knockdown. No significant differences in cell viability and total numbers of cells were observed between control siRNA and hnRNP C1/C2-specific siRNA transfection (Figure 3). However, it should be noted that the total number of hnRNP C1/C2 knockdown cells seemed to be slightly lower than that of control cells at 48 h post-infection. These observations were in line with a previous report in HEK 293T cells showing that hnRNP C1/C2 knockdown did not enhance cell death but affected cell growth at 72 and 96 h post-culture, by impairment of cell cycle progression and accumulation of cells in the G2/M phase [37]. Therefore, it is possible that the consequences of hnRNP C1/C2 knockdown on the process of DENV replication may occur through direct interaction of hnRNP C1/C2 with DENV RNA and/or through their effects on other cellular activities. The role of hnRNP C1/C2 in DENV replication deserves further investigation to explore molecular mechanisms in detail. Future study may also be extended to other types of target cells, such as monocytes and macrophages in primary infection and antibody-dependent enhancement condition, to assess whether hnRNP C1/C2 have any effect on host cellular response to DENV infection.

## Conclusions

The present study using DENV-infected Huh7 cells in the presence of siRNA-specific gene knockdown demonstrated hnRNP C1/C2 association with viral RNA and a potential important role of the hnRNP C1/C2 in supporting DENV replication, most likely at the stage of viral RNA synthesis. Our findings pave the way for further study on the molecular mechanisms of the viral and host protein interactions required for viral replication in DENV-infected cells.

## Materials and methods

### Cell lines, virus, and antibodies

Human hepatocellular carcinoma (Huh7) cells were obtained from JCRB Cell Bank (Osaka, Japan) and cultured in RPMI 1640 (Gibco, Carlsbad, CA, USA) supplemented with 10% heat-inactivated fetal bovine serum (FBS; Gibco), 2 mM L-glutamine (Sigma, St. Louis, MO, USA), 1% non-essential amino acid (NEAA; Gibco), 37 μg/ml penicillin (Sigma) and 60 μg/ml streptomycin (Sigma) at 37°C in a 5% CO_2_ incubator with a humidified atmosphere. Propagation of DENV serotype 2 (strain 16681) was performed in C6/36 mosquito cells. Mouse monoclonal antibodies specific for DENV NS1 (clone NS1-3 F.1) and DENV E (clones 3H5 and 4G2) were produced from previously established hybridoma cells [41-43]. Mouse monoclonal antibodies specific for human hnRNP C1/C2 (clone 4 F4) and human β-actin (clone C4) were purchased from Santa Cruz Biotechnology (Santa Cruz, CA, USA). Mouse isotype-matched control IgG1 antibody (clone MOPC 21) was purchased from Sigma.

### Immunoprecipitation of hnRNP C1/C2 proteins

Huh7 cells (5 × 10^6^) were seeded into a T-75 cm^2^ flask (Costar, Cambridge, MA, USA) and cultured for 24 h. The adherent cells were incubated with DENV-2 at a multiplicity of infection (MOI) of 0.5 in the culture medium at 37°C in a 5% CO_2_ incubator for 2 h. The supernatant containing DENV was discarded and the culture was maintained in fresh medium under the conditions described above. At 48 h post-infection, uninfected cells (mock control) and DENV-infected Huh7 cells were harvested and clear lysates were prepared by resuspending cell pellets with RIPA buffer containing 20 mM Tris–HCl, pH 7.4, 5 mM EDTA, 150 mM NaCl, 1% Triton X-100, 0.1% SDS, and 0.5% deoxycholate with protease inhibitor cocktail (Roche, Mannheim, Germany), then incubated on ice for 30 min and centrifuged at 9100 *g* at 4°C for 5 min. The clear lysates were subjected to immunoprecipitation by incubating with 5 μg mouse isotype-matched control IgG1 antibody or mouse anti-hnRNP C1/C2 antibody (clone 4 F4, IgG1) at 4°C overnight. Thereafter, the samples were incubated with 50% slurry protein-G-conjugated sepharose 4B beads (GE Healthcare, Uppsala, Sweden) at 4°C for 2 h followed by centrifugation at 15,300 *g* at 4°C for 5 min, and three washes with RIPA buffer. The immunoprecipitated complexes were eluted with nonreducing buffer and subjected to immunoblotting analysis to determine the presence of hnRNP C1/C2 proteins. Alternatively, the immunoprecipitated complexes on the beads were directly processed for RNA extraction and subsequent reverse transcriptase polymerase chain reaction (RT-PCR) for determination of DENV RNA.

### siRNA-mediated knockdown of hnRNP C1/C2

Huh7 cells were seeded onto a 24-well plate in medium without antibiotics (maintenance medium) at a concentration of 9 × 10^4^ cells/well. Fifteen hours later when the cells reached ~50% confluence, the medium was replaced with fresh RPMI medium and the cells were transfected with duplex hnRNP C1/C2-specific siRNA (siGENOME SMARTpool Human HNRNPC M-011869-01; Dharmacon, Lafayette, CO, USA) or duplex irrelevant siRNA (Stealth RNAi negative control medium GC; Invitrogen, Carlsbad, CA, USA), which is designed to minimize sequence homology to any known vertebrate transcript, using Lipofectamine 2000 (Invitrogen) according to the manufacturer’s instructions. After 6 h incubation with siRNA (114 nM), the cells were supplemented with maintenance medium and incubated for a further 18 h. The second round of siRNA transfection was performed using a similar approach to that described above. To test the effects of hnRNP C1/C2 knockdown on DENV infection, cells that had been subjected to the second round of siRNA transfection for 6 h were incubated with DENV-2 at MOI 0.03 or maintenance medium (mock control) for 2 h. The cells were washed twice and cultured in maintenance medium. The time interval between initial transfection and DENV infection was 32 h. At 0, 12, 24, 36 and 48 h post-infection, mock-infected and DENV-infected cells and their culture supernatants were collected. The total number of viable cells was enumerated using trypan blue (Gibco) exclusion dye assay. The percentage of dead cells was assessed by staining with propidium iodide (PI) at a final concentration of 2 ng/μl for 15 min at 4°C and subsequent flow cytometric analysis. Aliquots of the cells were processed for immunoblotting analysis, real-time RT-PCR, and immunofluorescence staining, and the culture supernatants were subjected to a focus forming unit (FFU) assay as described below.

### Immunoblotting analysis

Clear lysates or immunoprecipitated samples prepared from mock-infected and DENV-infected cells that were untransfected or transfected with siRNA were mixed with 4× loading buffer [50 mM Tris–HCl (pH 6.8), 2% SDS, 0.1% bromophenol blue and 10% glycerol] with or without 5% β-mercaptoethanol and heated at 95°C for 5 min. Proteins in the samples were subjected to 10% SDS-PAGE and transferred to PVDF membranes (Millipore, Billerica, MA, USA) as previously described [26]. The membranes were incubated with 5% skim milk in PBS or in Tris-buffered saline with 0.05% Tween 20 (TBST) for 1 h to block nonspecific binding and then with mouse monoclonal antibodies specific for DENV NS1 (clone NS1-3 F.1), DENV E (clone 4G2), human hnRNP C1/C2 (clone 4 F4) and human β-actin (clone C4) at 4°C overnight. The membranes were washed three times with PBS or TBST and incubated with HRP-conjugated rabbit anti-mouse immunoglobulin antibody (DAKO) at a dilution of 1:1000 for 1 h at room temperature, followed by a further three washes. Immunoreactive proteins were visualized using an enhanced chemiluminescence detection kit (Western Lightning Chemiluminescence Reagent Plus; Perkin Elmer Applied Biosystems, Foster City, CA, USA). Relative levels of human hnRNP C1/C2, DENV NS1 and DENV E protein expression were assessed by normalization of their protein band intensities to human β-actin intensity using GeneTools software from Syngene (Cambridge, UK).

### Determination of DENV RNA in immunoprecipitated complexes

RNA was extracted from immunoprecipitated complexes captured on protein G sepharose beads by TRIzol reagent (Invitrogen) and subjected to reverse transcription using SuperScript III Reverse Transcriptase (Invitrogen) according to the manufacturer’s protocol, with the NS1-R primer (Table 1). The resultant cDNA was used as a template for amplification of DENV NS1 region with the NS1-F and NS1-R primers (Table 1) using Biometra TGradient Thermal Cycler (Biometra GmbH, Goettingen, Germany). A 25-μl reaction contained cDNA template, 1× Green GoTaq Flexi Reaction Buffer (Promega, Madison, WI, USA), 2.5 mM MgCl_2_, 0.2 mM dNTPs, and 0.4 μM primers NS1-F and NS1-R, 0.5 U Taq DNA polymerase (Promega) in deionized water. The PCR reaction was preheated at 94°C for 5 min and processed through 35 cycles of denaturation (94°C, 30 s), annealing (48°C, 30 s) and extension (72°C, 1 min 30 s), as well as a final extension at 72°C for 10 min. The PCR products were electrophoresed in 1.5% SeaKem LE Agarose gel (Cambrex Bio Science; Rockland, ME, USA) and visualized by Gene Genius Bio Imaging system (Syngene).

**Table 1:** **Oligonucleotide primers for reverse transcription-PCR**

**Primer**	**Orientation**	**Sequence**
NS1-F	Sense	5′ CCGGCCAGATCTGATAGTGGTTGCGTTGTGAGC 3′
NS1-R	Antisense	5′ GATCGATCGCGGCCGCTTAGGCTGTGACCAAGG AGTTAACCAAATTCTCTTCTTTCTC 3′
hnRNP C1/C2-F	Sense	5′ TCGAAACGTCAGCGTGTATC 3′
hnRNP C1/C2-R	Antisense	5′ TCCAGGTTTTCCAGGAGAGA 3′
DEUR	Antisense	5′ GCTGTGTCACCCAGAATGGCCAT 3′
D2L	Sense	5′ ATCCAGATGTCATCAGGAAAC 3′
D2R	Antisense	5′ CCGGCTCTACTCCTATGATG 3′
actin-F	Sense	5′ AGAAAATCTGGCACCACAAA 3′
actin-R	Antisense	5′ CTCCTTAATGCTACGCACGA 3′

### Real-time RT-PCR for measurement of hnRNP C1/C2 mRNA and DENV RNA

RNA was extracted from DENV-infected cells that were transfected with irrelevant siRNA or hnRNP C1/C2-specific siRNA by TRIzol reagent (Invitrogen). Reverse transcription was performed using 62.5 ng total RNA and SuperScript III Reverse Transcriptase (Invitrogen) or AMV Reverse Transcriptase (Promega), according to the manufacturer’s instructions with minor modifications. Oligo(dT) 20 primer and DEUR primer (Table 1) were used to synthesize cDNA templates for determination of human hnRNP C1/C2 and β-actin mRNA as well as DENV RNA, respectively. The resultant cDNA was used as a template for real-time PCR according to the manufacturer’s instructions for Light Cycler 480 SYBR Green I master mix (Roche, Mannheim, Germany), using primer pairs specific for human hnRNP C1/C2 and β-actin as well as DENV E (D2L and D2R) (Table 1). Real-time PCR was performed by LightCycler 480 II (Roche) with: (i) pre-incubation at 95°C for 10 min; (ii) 45 amplification cycles of denaturation at 95°C for 10 s, annealing at 62°C for 10 s, and extension at 72°C for 20 s; and (iii) melting curve and cooling steps as recommended by the manufacturer. Relative levels of human hnRNP C1/C2 mRNA expression were determined by normalization to the expression levels of human actin according to the 2^–ΔΔCt^ method [44]. Standard DENV RNA with known concentration (copy number/μl) that was subjected to the same reverse transcription and real-time PCR processes was used as a control for determining the amount of viral RNA in DENV-infected cells.

### Immunofluorescence staining for the detection of DENV E antigen

To determine the percentage of DENV infection, mock-infected and DENV-infected cells that were transfected with irrelevant siRNA or hnRNP C1/C2-specific siRNA were fixed with 4% paraformaldehyde in PBS, smeared on a glass slide, and left to air dry for 30 min at room temperature. The cells were permeabilized with 0.2% Triton X-100 for 10 min at room temperature and incubated successively with mouse anti-DENV E monoclonal antibody (clone 3H5) for 1 h at room temperature and Alexa-Fluor-488-conjugated goat anti-mouse IgG antibody (Invitrogen) at a dilution of 1:1,000 for 30 min at room temperature. The stained cells were visualized by a confocal laser-scanning microscope (LSM 510 Meta, Carl Zeiss, Jena, Germany).

### Viral translation assay

A DENV-2 reporter construct was generated by cloning T7 promoter, DENV-2 5′-UTR, the first 72 nucleotides of DENV-2 capsid-coding sequence, firefly luciferase gene and DENV-2 3′-UTR into pGL3-Basic (Promega) according to a previously described method [45]. Resultant viral reporter construct (namely pGL3-DENV2-5′UTR-72ntC-Fluc-3′UTR) and pRL-SV40 vector (Promega), which contains *Renilla* luciferase gene and serves an internal control reporter construct, were linearized with *Xba*I. One μg of purified DNA was subjected to *in vitro* transcription using the RiboMAX Large Scale RNA Production System-T7 (Promega) in the presence of 20 mM m^7^G(5′)ppp(5′)G RNA cap structure analog (New England BioLabs, Ipswich, MA, USA) and resultant RNA product was purified using RNeasy Mini Kit (QIAGEN, Hilden, Germany). To determine the effect of hnRNP C1/C2 knockdown on DENV translation, Huh7 cells were transfected twice with hnRNP C1/C2-specific siRNA or control siRNA (286 nM each) in a 96-well plate within a 24-h interval using Lipofectamine RNAiMAX (Invitrogen) according to the manufacturer’s instructions. After the second round of siRNA transfection, cells were co-transfected with 21 nM DENV reporter RNA and 2.1 nM control reporter RNA using Lipofectamine 2000 (Invitrogen) followed by replacement with fresh culture medium at 4 h later. Following 12 h after transfection with viral and control reporter RNA, cells were harvested and determined for firefly and *Renilla* luciferase expression using Dual-Luciferase Reporter Assay System (Promega). Both firefly and *Renilla* luciferase signals were measured by Synergy H1 Hybrid multi-mode microplate reader (BioTek, Winooski, VT, USA). Cells that had been similarly processed in the absence of reporter RNA served as background controls for luciferase assay. Additionally, reporter RNA-transfected cells that were treated with 286 nM DENV capsid-specific siRNA known to reduce the production of viral protein and infectious virus [46] or with 50 μM cycloheximide, an inhibitor of protein translation, were included in the assay to serve as positive controls for inhibition of protein translation.

### Focus forming unit (FFU) assay for the determination of DENV production

Supernatants collected from cultured DENV-infected cells that had been transfected with irrelevant siRNA or hnRNP C1/C2-specific siRNA were assessed for the production of infectious DENV. Vero cells were seeded onto a 96-well plate (Costar) at 2.5 × 10^4^ cells/well in minimal essential medium (MEM) supplemented with 10% FBS, 2 mM L-glutamine, 36 μg/ml penicillin and 60 μg/ml streptomycin, and cultured at 37°C in a 5% CO_2_ incubator for 24 h to obtain ~90% confluence of the cell monolayer. The medium was removed from each well. DENV was serially diluted 10-fold in MEM containing 3% FBS, 2 mM L-glutamine, 36 μg/ml penicillin and 60 μg/ml streptomycin, added to each well (100 μl/well) and incubated at 37°C in a 5% CO_2_ incubator for 2 h. Overlay medium (MEM containing 3% FBS, 10% tryptose phosphate broth and 1.5% gum tragacanth) was added to each well (100 μl/well), and the culture was incubated for a further 3 days under the same conditions. On the third day post-infection, the medium was discarded from DENV-infected cells and the adherent cells were washed three times with PBS (pH 7.4). The cells were fixed with 3.7% formaldehyde (BDH Laboratory Supplies, Poole, UK) in PBS at room temperature for 10 min followed by an additional 10 min of permeabilization with 1% Triton X-100 (Fluka, Steinheim, Switzerland). The cells were incubated sequentially with mouse anti-DENV E monoclonal antibody (clone 4G2) at room temperature for 1 h and HRP-conjugated rabbit anti-mouse Igs (DAKO) at a dilution of 1:500 in PBS containing 2% FBS and 0.05% Tween-20 in the dark at room temperature for 30 min. To develop an enzymatic reaction, the cells were incubated with a substrate solution containing 0.6 mg/ml diaminobenzidine (DAB), 0.03% H_2_O_2_ and 0.08% NiCl_2_ in PBS at room temperature in the dark for 5 min. After three washes with PBS, dark brown foci of the DENV-infected cells were counted under a light microscope. Virus titers were reported as FFU/ml from the duplicated samples.

### Statistical analysis

Relative levels of hnRNP C1/C2 mRNA and protein expression, cell viability, cell proliferation, percentage DENV infection, amount of DENV RNA, relative levels of DENV NS1 and E proteins, relative luciferase expression, and titers of infectious DENV were statistically analyzed by unpaired *t* test, with the use of GraphPad Prism version 5.0. Results were expressed as mean and standard error of the mean (SEM) and p < 0.05 was considered significant.
