# Correction: Role of human heterogeneous nuclear ribonucleoprotein C1/C2 in dengue virus replication

**DOI:** 10.1186/s12985-024-02309-x

**Published:** 2024-02-15

**Authors:** Thanyaporn Dechtawewat, Pucharee Songprakhon, Thawornchai Limjindaporn, Chunya Puttikhunt, Watchara Kasinrerk, Sawanan Saitornuang, Pa-thai Yenchitsomanus, Sansanee Noisakran

**Affiliations:** 1 Division of Molecular Medicine, Office of Research and Development, Faculty of Medicine Siriraj Hospital, Mahidol University, 10700 Bangkok, Thailand; 2 Graduate Program in Immunology, Department of Immunology, Faculty of Medicine Siriraj Hospital, Mahidol University, 10700 Bangkok, Thailand; 3 Department of Anatomy, Faculty of Medicine Siriraj Hospital, Mahidol University, 10700 Bangkok, Thailand; 4 Medical Biotechnology Research Unit, National Center for Genetic Engineering and Biotechnology, National Science and Technology Development Agency, 10700 Bangkok, Thailand; 5 Division of Dengue Hemorrhagic Fever Research Unit, Office of Research and Development, Faculty of Medicine Siriraj Hospital, Mahidol University, 10700 Bangkok, Thailand; 6 Division of Clinical Immunology, Department of Medical Technology, Faculty of Associated Medical Sciences, Chiang Mai University, 50200 Chiang Mai, Thailand; 7 Biomedical Technology Research Center, National Center for Genetic Engineering and Biotechnology, National Science and Technology Development Agency, 50200 Chiang Mai, Thailand

**Correction: Virology Journal (2015) 12:14**

10.1186/s12985-014-0219-7

Following publication of the original article [1], the authors informed us that the citation number 5., “Recent advances in DENV receptors, The Scientific World Journal, Volume 2013,” was excluded from the original article due to its retraction in 2013.
